# Supplementary material for: The UK Chinese population with kidney failure: Clinical characteristics, management and access to kidney transplantation using 20 years of UK Renal Registry and NHS Blood and Transplant data
Source: PLoS One. 2022 Feb 28;17(2):e0264313. doi: 10.1371/journal.pone.0264313 (PMC8884499; doi:10.1371/journal.pone.0264313)
Supplement: S1 Table — (DOCX) [file pone.0264313.s001.docx]

| **Variable** | **Missing data**  **from complete dataset**  **n (%)** | **Missing data for Chinese patients**  **n (%)** | **Missing data for White patients**  **n (%)** | **Notes** |
| --- | --- | --- | --- | --- |
| Ethnicity | 4918 (5.3) | - | - | Ethnicity data was more likely to be missing by country (chi2 p<0.001), and centre (P<0.001) |
| Age | 0 (0) | 0 (0) | 0 (0) |  |
| Sex | 0 (0) | 0 (0) | 0 (0) |  |
| IMD | 402 (0.4) | 1 (0.2) | 283 (0.4) |  |
| Country | 402 (0.4) | 1 (0.2) | 283 (0.4) |  |
| Primary renal diagnosis | 3680 (4) | 2239 (3) | 19 (4) |  |
| ERA-EDTA primary renal diagnosis code (pre-2012) | 3680 (4) | 2239 (3) | 19 (4) |  |
| Revised ERA-EDTA primary renal diagnosis code (2012 onwards) | 27996 (30) | 119  (24) | 20,868 (30) | More White KRT patients were missing a revised ERA-EDTA primary renal diagnosis code than UK Chinese patient (Chi2 P=0.009) |
| Modality at start of KRT | 0 (0) | 0 (0) | 0 (0) |  |
| Co-morbidity | 56,659 (61) | 305  (62) | 43,259 (61) | There was no evidence of difference in proportion of missing data for this variable between UK Chinese and White patients (Chi2 P=0.756) |
| Body mass index | 64,087 (69) | 340 (69) | 48,615 (69) | There was no evidence of difference in proportion of missing data for the BMI variable between UK Chinese and White patients (Chi2 P=0.921) |
