# Supplementary material for: The UK Chinese population with kidney failure: Clinical characteristics, management and access to kidney transplantation using 20 years of UK Renal Registry and NHS Blood and Transplant data
Source: PLoS One. 2022 Feb 28;17(2):e0264313. doi: 10.1371/journal.pone.0264313 (PMC8884499; doi:10.1371/journal.pone.0264313)
Supplement: S2 Table — (DOCX) [file pone.0264313.s002.docx]

**S2 Tables: Competing Risk Analyses**

**Table S2A: Sub-distribution hazard ratios investigating the association between Chinese ethnicity and access to wait-listing and transplant at 3 years, competing risk of death before wait-listing or transplantation**

|  | **Competing risk** | **Chinese vs White**  **SHR**  **CI, P-value*** | **Chinese vs White**  **SHR**  **CI, P-value**** | **Chinese vs White**  **SHR**  **CI, P-value***** |
| --- | --- | --- | --- | --- |
| Waitlisting at 2 years | Death before 2 years & death before listing | 1.28 [1.08-1.51] P=0.004 | 1.19 [1.00-1.41] P=0.05 | 1.19 [1.00-1.41] P=0.05 |
| Transplant at 3 years | Death before 3 years & death before listing | 0.78 [0.62-0.98] P=0.032 | 0.71 [0.56-0.90]  P=0.004 | 0.71 [0.56-0.90]  P=0.004 |

*** unadjusted analysis**

**** adjusted for age group, sex, socioeconomic status**

***** adjusted for age group, sex, socioeconomic status, primary renal disease**

**Table S2B: Sub-distribution hazard ratios investigating the association between Chinese ethnicity and access to wait-listing and transplant at 3 years, competing risk of death before wait-listing or transplantation, stratified by Sex**

|  | **Competing risk** | **Chinese vs White**  **SHR**  **CI, P-value*** | **Chinese vs White**  **SHR**  **CI, P-value**** | **Chinese vs White**  **SHR**  **CI, P-value***** |
| --- | --- | --- | --- | --- |
| **Male** | | | | |
| Waitlisting at 2 years | Death before 2 years & death before listing | 1.29 [1.05-1.59]  P=0.014 | 1.17 [0.94-1.46]  P=0.15 | 1.17 [0.94-1.46]  P=0.16 |
| Transplant at 3 years | Death before 3 years & death before listing | 0.69 [0.51-0.94]  P=0.02 | 0.61 [0.45-0.84]  P=0.002 | 0.61 [0.45-0.84]  P=0.002 |
| **Female** | | | | |
| Waitlisting at 2 years | Death before 2 years & death before listing | 1.26 [0.96-1.65]  P=0.10 | 1.21 [0.92-1.60]  P=0.17 | 1.21 [0.92-1.60]  P=0.18 |
| Transplant at 3 years | Death before 3 years & death before listing | 0.92 [0.66-1.29]  P=0.64 | 0.87 [0.61-1.23] P=0.42 | 0.87 [0.61-1.23]  P=0.43 |

*** unadjusted analysis**

**** adjusted for age group, socioeconomic status**

***** adjusted for age group, socioeconomic status, primary renal disease**

**Table S2C: Proportional cause specific hazards model investigating the association between Chinese ethnicity and access to wait-listing and transplant at 3 years, competing risk of death before wait-listing or transplantation**

|  |  | **Chinese vs White**  **HR**  **CI, P-value *** | **Chinese vs White**  **HR**  **CI, P-value **** | **Chinese vs White**  **HR**  **CI, P-value ***** | **Chinese vs White**  **HR**  **CI, P value ****** |
| --- | --- | --- | --- | --- | --- |
| Waitlisting at 2 years | Competing risk censored- death before 3 years and before waitlisting | 1.25  [1.07-1.49]  P=0.007 | 1.26  [1.07-1.48]  P=0.006 | 1.18  [0.97-1.41]  P=0.09 | 1.18  [0.97-1.42]  P=0.09 |
| Transplant at 3 years | Competing risk censored -death before 3 years and before transplant | 0.78  [0.62-0.98]  P=0.03 | 0.78  [0.61-0.99]  P=0.04 | 0.70  [0.56-0.89]  P=0.003 | 0.70  [0.56-0.89]  P=0.003 |

*** unadjusted analysis**

****robust standard errors clustered by centre**

***** adjusted for age group, socioeconomic status**

****** adjusted for age group, socioeconomic status, primary renal disease**

**Table S2D: Proportional cause specific hazards model investigating the association between Chinese ethnicity and access to wait-listing and transplant at 3 years, competing risk of death before wait-listing or transplantation, stratified by Sex**

|  |  | **Chinese vs White**  **HR**  **CI, P-value *** | **Chinese vs White**  **HR**  **CI, P-value **** | **Chinese vs White**  **HR**  **CI, P-value ***** | **Chinese vs White**  **HR**  **CI, P value ****** |
| --- | --- | --- | --- | --- | --- |
| **Male** | | | | | |
| Waitlisting at 2 years | Competing risk censored- death before 3 years and before waitlisting | 1.28  [1.03-1.58] P=0.02 | 1.28  [1.00-1.62]  P=0.05 | 1.17  [0.91-1.51]  P=0.23 | 1.17  [0.90-1.51]  P=0.24 |
| Transplant at 3 years | Competing risk Censored -death before 3 years and before transplant | 0.69 [0.50-0.94]  P=0.02 | 0.69 [0.49-0.96]  P=0.03 | 0.61 [0.44-0.83]  P=0.002 | 0.61 [0.44-0.83] P=0.002 |
| **Female** | | | | | |
| Waitlisting at 2 years | Competing risk censored- death before 3 years and before waitlisting | 1.23 [0.95-1.60]  P=0.12 | 1.23 [0.93-1.64]  P=0.15 | 1.18 [0.90-1.55]  P=0.22 | 1.18 [0.90-1.56]  P=0.24 |
| Transplant at 3 years | Competing risk Censored -death before 3 years and before transplant | 0.92 [0.65-1.29]  P=0.62 | 0.92 [0.67-1.26]  P=0.59 | 0.86 [0.61-1.2]  P=0.38 | 0.86 [0.61-1.20]  P=0.39 |

*** unadjusted analysis**

****robust standard errors clustered by centre**

***** adjusted for age group, socioeconomic status**

****** adjusted for age group, socioeconomic status, primary renal disease**
