# Supplementary material for: The UK Chinese population with kidney failure: Clinical characteristics, management and access to kidney transplantation using 20 years of UK Renal Registry and NHS Blood and Transplant data
Source: PLoS One. 2022 Feb 28;17(2):e0264313. doi: 10.1371/journal.pone.0264313 (PMC8884499; doi:10.1371/journal.pone.0264313)
Supplement: S3 Table — (DOCX) [file pone.0264313.s003.docx]

**S3 Table:** **Results of multivariable logistic regression analyses investigating Chinese ethnicity and access to wait-listing and transplantation, stratified by Sex**

|  | **Unadjusted analysis for Females**  **Chinese females vs White females**  **OR, (95% CI),**  **P-value** | **Adjusted* analysis for Females**  **Chinese females vs White females**  **OR, (95% CI),**  **P-value** | **Unadjusted analysis for Males**  **Chinese Males vs White Males**  **OR, (95% CI),**  **P-value** | **Adjusted* analysis for Males**  **Chinese Males vs White Males**  **OR, (95% CI),**  **P-value** |
| --- | --- | --- | --- | --- |
| Waitlisting at start of KRT | 0.87 [0.59-1.30]  P=0.50 | 0.85 [0.54-1.34]  P=0.49 | 0.71 [0.49-1.0]  P=0.06 | 0.66 [0.43-1.00]  P=0.05 |
| Waitlisting within 2 years of KRT start | 1.39 [1.02-1.90]  P=0.04 | 1.46 [1.09-1.96]  P=0.01 | 1.23 [0.95-1.60]  P=0.11 | 1.13 [0.84-1.56]  P=0.41 |
| Pre-emptive transplant | 0.80 [0.44-1.44]  P=0.46 | 0.78 [0.38-1.61]  P=0.50 | 0.30 [0.14-0.68]  P=0.004 | 0.28 [0.10-0.76]  P=0.01 |
| Transplanted within 3 years of KRT start | 0.97 [0.69-1.37]  P=0.85 | 0.94 [0.60-1.46]  P=0.78 | 0.60 [0.43-0.82]  P=0.002 | 0.50 [0.34-0.73]  P<0.001 |
| LDKT | 0.31 [0.16-0.60]  P<0.001 | 0.32 [0.18-0.57]  P<0.001 | 0.36 [0.20-0.64]  P<0.001 | 0.35 [0.20-0.61]  P<0.001 |
| ***Adjusted for age, primary renal disease, and socioeconomic status** | | | | |
