# Supplementary material for: The UK Chinese population with kidney failure: Clinical characteristics, management and access to kidney transplantation using 20 years of UK Renal Registry and NHS Blood and Transplant data
Source: PLoS One. 2022 Feb 28;17(2):e0264313. doi: 10.1371/journal.pone.0264313 (PMC8884499; doi:10.1371/journal.pone.0264313)
Supplement: S1 File — (PDF) [file pone.0264313.s004.pdf]

NHS Blood and Transplant

Consent for Organ and/or Tissue Donation

Unique Tissue Number K

ODT Donor number

COMPLETE ONLY WHEN OBTAINING **CONSENT VIA TELEPHONE** — Section 1

The person taking consent **must** ask the following questions and **initial** the appropriate boxes (it is not a legal requirement for the consent form to be signed by the family):

	Yes	No
Do you agree to the conversation about donation between (name of Healthcare Professional) of NHS Blood and Transplant and you being voice recorded? The recording will be stored as proof of the information that I give to you and of the consent and information that you give to me.		
For the purpose of the recording can you tell me again your full name and relationship to (name of the patient).		
May we use the recording and case details for our records?		

PATIENT DETAILS — Section 2

Name

Address

Postcode

NHS number

Hospital number

Date of birth

Age years months
(If under 3 years record years and months)

CONSENT FOR ORGANS AND TISSUE — Section 3

Complete **Section A** if the patient is giving/has given **first person consent**
OR
Complete **Section B** if consent is given by **nominated/appointed representative** or the **person ranking highest in the qualifying relationship**
OR
Complete **Section C** if consent is able to be **deemed** in accordance with the appropriate legislation *****

Section A

The patient named in **Section 2** gave/gives* first person consent for the donation of the following organs/tissue for transplantation via the Organ Donor Register/donor card/expressed wish/will* *(*Delete as appropriate)*

Healthcare Professional signature

Section B

(Name) the **(Relationship to the patient)** of **(Patient's name)**

Gives consent for the donation of the following organs/tissue for transplantation as detailed on page 2 of this form.

	Yes	No
Did the patient have a nominated/appointed representative?		
Was consent obtained from the person ranking highest in the qualifying relationship?		

If no, please give details below:

Persons are ranked in the following descending order:
A) spouse or partner (including civil or same sex partner)
B) parent or child
C) brother or sister
D) grandparent or grandchild
E) niece or nephew
F) stepfather or stepmother
G) half-brother or half-sister
H) friend of long standing
Jersey only – before (h) For person of whom a care order is made: - the Minister for Health and Social Services

Section C
For Countries and Territories where Deemed Consent applies

The patient named in **Section 2** is deemed to have given their consent to deceased organ and/or tissue donation under the applicable legislation*.

Please insert Country/Territories ……………………….

Healthcare Professional signature

NHS Blood and Transplant

Consent for Organ and/or Tissue Donation

Unique Tissue Number K

ODT Donor number

CONSENT FOR DONATION

Section 4

Please initial appropriate box

	Yes	No	Exclusion	Coroner Restriction
Kidneys				
Liver				
Liver for Hepatocytes				
Pancreas				
Pancreas for Islet Cells				
Heart				
Heart for Valves				
Lungs				
Bowel				
Multivisceral*				
*If yes, please specify explicitly				
Other**				
**If yes, please specify explicitly				
Eyes				
Skin				
Bone				
Tendons (Ankle & Knee)				
Femoral Artery				
Meniscus				
Other Tissue***				
***If yes, please specify explicitly				
Blood Vessels (see core information)				

ONLY USE IN CIRCUMSTANCES WHEN FAMILIES DO NOT WANT INFORMATION

All Abdominal Organs (including Liver for Hepatocytes, Pancreas for Islet cells)

Yes No

Blood Vessels (see core information)

All Cardiothoracic Organs

Yes No

All Tissues (excluding Liver for Hepatocytes, Pancreas for Islet cells)

Yes No

NATIONAL REFERRAL CENTRE USE ONLY

All Tissues

Yes No

Do you consent to the patient being transferred from his/her place of death to the NHSBT Tissue services donation facility or an alternative establishment i.e. another hospital mortuary for the donation procedure to be undertaken, if applicable?

Yes **No** **N/A**

Consent for Organ and/or Tissue Donation

NHS Blood and Transplant

Unique Tissue Number K

ODT Donor number

CONSENT FOR SCHEDULED PURPOSES — Section 5

Reminder - Deemed Consent does not apply to Scheduled Purposes

Organs and/or tissue can also be used for the Scheduled Purposes* listed below:

Scheduled Purposes Include:
Research
Education or Training related to Human Health
Clinical audit
Quality Assurance
Performance Assessment

*only relevant Scheduled Purposes listed.

1. There is also an opportunity to support transplantation/healthcare through the removal of samples, for example blood, urine and/or tissue samples from specific organs which can then be used in approved research projects. Do you believe the patient would agree to this **and** do you consent? **Yes** ☐ **No** ☐ **N/A** ☐

2. On occasion, organs/tissues you have agreed to donate may be found to be unsuitable when removed for transplant. However, these organs/tissues can be used in research (or other Scheduled Purposes as detailed above) to improve healthcare in the future, do you consent to this? **Yes** ☐ **No** ☐

3. Organs/tissues/samples may also be donated and used to improve future healthcare.
Do you consent to the removal and storage of specific organ/tissues/samples for research or other Scheduled Purposes as detailed above?

A. In QUOD-Licensed Hospitals Only

	Yes	No	N/A
Heart	☐	☐	☐
Lungs	☐	☐	☐
Diabetic Pancreas	☐	☐	☐
B. Centre-Licensed Specific Studies	☐	☐	☐

(For questions 1 & 3B - Please detail specific organ/tissue/samples in notes on page 6).

Any research or other Scheduled Purposes restriction? Yes ☐ No ☐ N/A ☐

If yes, please provide detail

Organs and/or tissue will be used and stored for a Scheduled Purpose in accordance with The Human Tissue Act (2004)

Organs and/or tissue and/or material that are not used will be disposed of as per local establishment policy in accordance with the Human Tissue Act (2004).

Consent for Organ and/or Tissue Donation

NHS Blood and Transplant

Unique Tissue Number	K								

ODT Donor number						

SHARING OF INFORMATION REQUIRED TO SUPPORT ORGAN AND TISSUE DONATION — Section 6

Core information
Blood samples will be obtained from the patient for testing, including pregnancy (for organ donors only, if applicable), tissue typing, HIV, Hepatitis, HTLV and Syphilis. These samples may be subsequently stored for future testing as necessary. In the event of any screening results that may have implications for the family, relevant individuals may be contacted if their health could be affected.

For paediatric donation only:
Blood samples will be obtained from the patient and the patient's mother where the patient is under 18 months old and/or the patient has been breast fed in the last 12 months for testing, including tissue typing, HIV, Hepatitis, HTLV and Syphilis. These samples may be subsequently stored for future testing as necessary. In the event of any screening results that may have implications for the family, relevant individuals may be contacted if their health could be affected.

Organ donation:
Tissue samples for example, lymph node and spleen that have been obtained for screening will be subsequently biopsied, analysed and stored for future testing as necessary.
Blood vessels will be retrieved and stored to support organ transplant or other surgical procedures, if not used within 14 days will be disposed of in accordance with the hospital/tissue establishment policy.

Tissue donation:
The tissue donated (including hepatocytes and heart for valves) for transplantation will be stored for extended periods in tissue establishments whilst it is prepared for transplantation.

The patient's medical records have/will be accessed by relevant healthcare professionals to obtain a past medical/behavioural history. This information may be passed on a need-to-know basis to other healthcare professionals in support of the donation and transplantation process. This information may also be retained by the Organ Donation Teams/Tissue Establishment.
The information collected from you on the consent form will be stored securely and only be used in support of the donation and transplantation process. For further information please see the privacy statement on NHSBT's website:
https://www.nhsbt.nhs.uk/privacy/

Core information has been provided

☐

Applicable legislation:
Consent is obtained in accordance with the following legislation and good practice guidance:

Human Tissue Act (2004)
Human Transplantation (Wales) Act 2013 *
Human Transplantation and Anatomy (Jersey) Law 2018 *
The Human Tissue Authority Codes of Practice
Organ Donation (Deemed Consent) Act 2019 *
The Human Tissue Authority Code of Practice for organ donation to support the Human Transplantation (Wales) Act 2013
Mental Capacity Act (2005)
Mental Capacity Act (Northern Ireland) 2016
General Data Protection Regulation 2016

Consent for Organ and/or Tissue Donation

NHS Blood and Transplant

Unique Tissue Number K

ODT Donor number

CONFIRMATION OF CONSENT

Section 7

I have understood the above and I have had the opportunity to ask questions which have been answered to my satisfaction.

Patient/Relationship to patient

Name Please print

Signed

Date 2 0

Time *(24hr)* :

Address of person giving consent

Email address

Telephone number

Mobile

Co-signatory Name *(Where applicable)* Please print

Signed

Relationship to patient

Email address

Telephone number/mobile

Healthcare Professional Details (Witness)

Designation

Please print

Name

Signed

Date 2 0

Time *(24 hr)* :

Healthcare Professional Ascertaining Consent

Designation

Please print

Name

Signed

Date 2 0

Time *(24 hr)* :

Information leaflets given to family? ☐ **OR** **Information leaflets to be sent to the family?** ☐

Consent for Organ and/or Tissue Donation

NHS Blood and Transplant

Unique Tissue Number	K								

ODT Donor number						

Additional Information

Consent for Organ and/or Tissue Donation

NHS Blood and Transplant

Unique Tissue Number	K								

ODT Donor number						

Additional Information
